# HTLV-1, Immune Response and Autoimmunity

**DOI:** 10.3390/v8010005

**Published:** 2015-12-24

**Authors:** Juarez A S Quaresma, Gilberto T Yoshikawa, Roberta V L Koyama, George A S Dias, Satomi Fujihara, Hellen T Fuzii

**Affiliations:** 1 Science Center of Health and Biology. Pará State University, Rua Perebebuí, 2623, Belém, Pará 66087-670, Brazil; juarez@ufpa.br (J.A.S.Q.); robertakoyamareumato@gmail.com (R.V.L.K.); georgealbertodias@yahoo.com.br (G.A.S.D.); 2 Science Health Institute, Federal University of Pará, Praça Camilo Salgado, 1, Belém, Pará 66055-240, Brazil; gyoshikawa@uol.com.br; 3 Tropical Medicine Center, Federal University of Pará, Av. Generalíssimo Deodoro, 92, Belém, Pará 66055-240, Brazil; satomifujihara@gmail.com

**Keywords:** Human T-lymphotropic virus type-1 (HTLV-1), immune response, autoimmunity

## Abstract

Human T-lymphotropic virus type-1 (HTLV-1) infection is associated with adult T-cell leukemia/lymphoma (ATL). Tropical spastic paraparesis/HTLV-1-associated myelopathy (PET/HAM) is involved in the development of autoimmune diseases including Rheumatoid Arthritis (RA), Systemic Lupus Erythematosus (SLE), and Sjögren’s Syndrome (SS). The development of HTLV-1-driven autoimmunity is hypothesized to rely on molecular mimicry, because virus-like particles can trigger an inflammatory response. However, HTLV-1 modifies the behavior of CD4^+^ T cells on infection and alters their cytokine production. A previous study showed that in patients infected with HTLV-1, the activity of regulatory CD4^+^ T cells and their consequent expression of inflammatory and anti-inflammatory cytokines are altered. In this review, we discuss the mechanisms underlying changes in cytokine release leading to the loss of tolerance and development of autoimmunity.

## 1. Introduction

The etiology of autoimmune diseases is unknown, but it is clear that the interaction between genes and the environment is an important step in breaking immune tolerance to self-antigens. This can lead to inflammation and destruction of specific tissues and organs. Although host genetic background contributes to autoimmunity, research indicates that infectious agents are one of most important environmental factors responsible for the development of autoimmune diseases [1,2,3]. Chronic inflammatory responses to infections have been associated with the initiation and exacerbation of autoimmune diseases [1,4,5].

Human T-lymphotropic virus type-1 (HTLV-1) is associated to a number of diseases, such as HTLV-1-associated myelopathy/tropical spastic paraparesis (HAM/TSP), and autoimmune diseases such as the Sjögren’s Syndrome (SS), arthropathies, and uveitis, which are often related to changes in the immune response [6,7,8,9]. The HTLV-1 virus infects CD4^+^ T lymphocytes, and can modify the cell function. CD4^+^ T lymphocytes are the central acquired immune response regulators. Changes in their behavior can trigger inflammatory reactions that can break immune system tolerance, leading to autoimmunity. In this review, we discuss immunological changes in HTLV-1 infection and its association with autoimmune diseases.

## 2. Human T-lymphotropic Virus Type-1 (HTLV-1)

Human T-lymphotropic virus type-1 (HTLV-1) is classified as a complex type C retrovirus belonging to the genus *Deltaretrovirus*, family *Retroviridae,* and subfamily *Orthoretrovirinae* [10,11]*.*

The morphological structure of this virus is similar to other retroviruses; the capsid contains two simple RNA strands together with the reverse transcriptase and integrase enzymes. These enzymes are important for insertion of the virus into the host genome, resulting in a provirus [12,13,14,15].

The virus genome contains structural and functional genes, such as the *gag*, *pro/pol*, and *env* that are flanked by two long terminal repeat (LTR) regions. Additionally, the *pX* region was identified in the region between the *env* gene and the 3′-LTR region. The *pX* region codes for the Tax (*p*40), REX (*p*27), *p*12, *p*13, *p*21, and *p*30 regulatory proteins that are involved in viral infection and proliferation. Tax is a phosphoprotein that exerts an essential role in viral transcription and cell behavior transformation [16,17,18,19]. It has pleiotropic functions that occur on a very wide spectrum of interactions with cellular proteins. Tax can modify signal transduction pathways of the host–cell that induce the transcription factors NF-κB, cAMP response element binding (CREB), Serum response factor (SRF) and activator protein 1 (AP-1) [12,13,20,21,22].

Recently, another gene was identified, *hbz* (*HTLV-1 b-ZIP factor*), that codes for a protein involved in the pathogenesis of the virus together with Tax. HBZ can function in two different molecular forms, mRNA and protein. In mRNA form, it can promote cell proliferation by positive regulation of E2F1. HBZ protein can down-regulate Tax expression and can interact directly with c-Jun and c-Jun-B [23,24].

HTLV-1 infection is associated with several diseases, primarily with adult T-cell lymphoma (ATL) and HAM/TSP. HAM/TSP patients present a series of immunological dysfunctions, including spontaneous proliferation of HTLV-infected T CD4^+^ lymphocytes, an increase in the migratory capacity of circulating leukocytes, and increased production of inflammatory cytokines—particularly neurotoxic cytokines such as IFN-γ and TNF-α—in affected regions along the spinal cord [6,16,25,26,27,28]. In HAM/TSP patients, there is a predominance of Th1 cytokines (IFN-γ) and a reduction in Th2 cytokines (IL-4 and IL-10), this is likely to cause greater circulation of immune cells between peripheral blood and the central nervous system (CNS), leading to inflammation of the nervous tissue [29,30,31]. Among the several existing theories related to HAM/TSP development, the most widely accepted is that it is a virally induced, cytotoxic, demyelinating inflammatory process of a chronic and progressive nature. The lymphocytes are activated during spastic paraparesis; when they cross the blood–brain barrier, the inflammatory process initiates in the CNS, resulting in lesions [10,15,26,32,33]. Another theory is direct cytotoxicity mechanism, where HTLV-1-cytotoxic CD8^+^ T cell cross the blood-brain barrier and destroy HTLV-1 infected glia cells by cytotoxicity or cytokine production. The last theory suggests that autoimmunity mechanism can cause lesions by molecular mimicry. A host neuronal protein seems to be similar to Tax protein from the virus, which can cause immune cross-reaction, leading to CNS inflammation [6,10,34,35].

## 3. Immunological Changes in HTLV-1-Infected Patients

Patients infected with HTLV-1 may develop a number of associated diseases, such as HAM/TSP, or other autoimmune diseases such as the Sjögren’s Syndrome (SS), arthropathies, and uveitis, which are often related to changes in the immune response [7,8,9,35]. These changes may occur due to infection of the CD4^+^ T lymphocytes by HTLV-1.

HTLV-1-infected CD4^+^ T lymphocytes exhibit altered signaling cascades and transcription factor activation, leading to changes in cell behavior [7,8,9,16,36,37].

Many studies have reported that the HTLV-1 Tax protein affects several transcription factors including CREB/ATF, NF-κB, AP-1, SRF, and Nuclear factor of activated T-cells (NFAT), as well as a number of signaling cascades involving PDZ domain-containing proteins such as Rho-GTPases and Janus kinase (JAK)/signal transducer and activator of transcription (STAT), thus altering the transforming growth factor-β (TGF-β) cascades. These factors are involved in cell proliferation and activation, including expression of cytokines and activation of viral proteins [17,18,19,38,39,40,41].

The expression of forkhead/winged helix transcription factor (FOXP3), which is an important transcription factor, has also been reported to be altered in patients infected with HTLV-1. FOXP3 is an essential transcription factor for the differentiation, function, and homeostasis of regulatory T cells (Tregs). Irregularities in the expression of FOXP3 may lead to loss of immune tolerance and the probable development of autoimmune diseases [41,42].

Previous studies have demonstrated that an increase in FOXP3 expression in patients that developed ATL leads to an exacerbated Treg function, resulting in increased production of IL-10 and TGF-β, which in turn triggers the immunosuppression phenotype observed in these patients. In contrast, studies performed with patients that developed HAM/TSP showed a decrease in FOXP3 expression and in the production of the IL-10 and TGF-β cytokines responsible for suppression of the immune response [31,36,43,44,45]. This loss of suppressive function may lead to an exacerbation of the disease process, since inflammation is not controlled and the inflammatory process is perpetuated. A study performed by Yamano *et al.* [31] showed that persistent activation of the immune response, induced by Tax, in patients with HAM/TSP may be associated with a decrease in the expression of CD4^+^CD25^+^FOXP3^+^ T cells that possess a suppressive function and an accumulation of CD4^+^CD25^+^FOXP3^-^ T cells that can exacerbate the pathogenic process of HAM/TSP. The authors demonstrated that in patients with HAM/TSP, there was an increase in the sub-population of IFN-γ-producing T cells with a CD4^+^CD25^+^FOXP3^-^ phenotype and that this increase correlated with the clinical severity of HAM/TSP.

Changes in signaling pathways and transcription factor activation caused by viral proteins play an important role in modifying immune response homeostasis, resulting in a cytokine environment that may influence the immune cells phenotype. The effect of the environment may lead to autocrine and paracrine activation and thus influence T cell differentiation and homeostasis. IFN-γ can stimulate the production of Th1 cells, while the presence of IL-4 leads to the differentiation of Th2 cells. Several studies have shown that the presence of these cytokines triggers the activity of known suppressors of cytokine signaling (SOCS) family proteins, which are able to inhibit the recruitment of STATs or the activation of JAKs. SOCS play a role in the maturation, differentiation, and maintenance of T lymphocytes [46,47,48]. SOCS-1 was shown to inhibit the activation of pathways stimulated by IFN-γ and IL-4, whereas SOC3 is important for maintaining the Th2 phenotype. Previous studies performed with HAM/TSP patients have demonstrated increased SOC-1 and decreased SOC-3 levels, suggesting a tendency towards the Th1 response [49].

The HTLV-1-infected CD4^+^ T lymphocytes of HAM/TSP patients exhibit spontaneous proliferation, in addition to an increased production of proinflammatory cytokines such as IFN-γ, TNF-α, IL-1, and IL-6; neurotoxic cytokines such as IFN-γ and TNF-α, in particular, are found at high concentrations in the spinal fluid of HAM/TSP patients [9,16,50,51]. These findings demonstrate that infected individuals have an immune response characteristic of the Th1 phenotype (IFN-γ and TNF-α) and a decrease in the Th2 profile (IL-4 and IL-10) [30,31,45].

Toulza *et al.* [43] observed that individuals with HAM/TSP had similar IL-10 levels as healthy individuals and that the TGF-β levels were significantly lower compared to the control group; this in turn could result in a decrease in the Treg cell-mediated suppressive function and thus contribute to the exacerbation of inflammation.

## 4. HTLV-1 and Autoimmunity

HTLV-1 infection leads to changes in the systemic immune response even in asymptomatic patients [36,52,53,54,55,56,57]. The regulation of the immune response is carefully organized so that the organism suffers no damage and that homeostasis is maintained. This is ensured via clonal selection that occurs during lymphocyte development concomitant with the destruction of the autoreactive cells [58] and the development of Treg cells. Treg cells are able to inhibit the proliferation of T cells *in vitro* and regulate the activity of CD4^+^ and CD8^+^ T cells *in vivo*. There are two major types of Treg cells, naturally occurring cells and cells produced in the periphery [59].

During the development of autoimmunity, there is a loss of tolerance to self-antigens, causing an inflammatory response that attacks organs and tissues of the individual. The pathogenesis of autoimmune diseases is generally studied in the context of T helper cells and the balance between the Th1 and Th2 responses. Thus, it has been observed that some diseases such as Rheumatoid Arthritis (RA) fit the Th1 profile, in which a cell-mediated response occurs, while others such as Systemic Lupus Erythematosus (SLE) fit the Th2 profile, involving antibodies and immunocomplexes in their physiopathology [60]. However, during the development of autoimmunity, there is a combination of several factors that are not only immunological, but also genetic and environmental [61,62]. Of these environmental factors, infections are of great importance, since they act as a trigger for autoimmunity [63,64].

**Figure 1:**
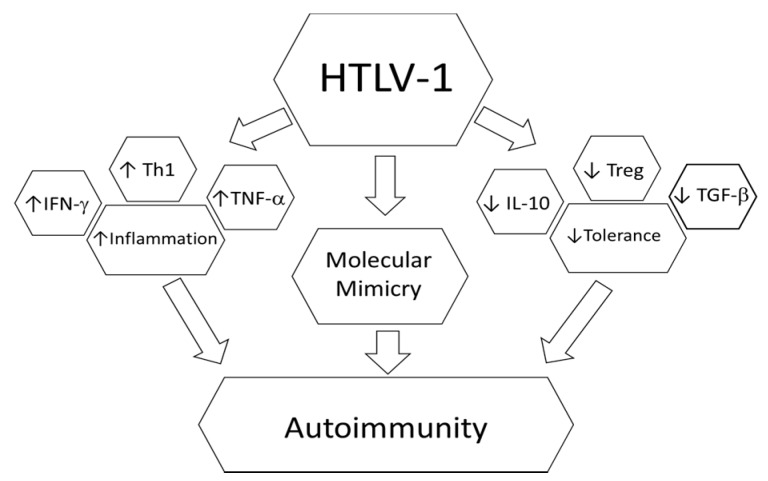
Possible mechanisms involved in HTLV-1 association with autoimmunity.

The association between autoimmunity and HTLV-1 infection has been previously described; however, the mechanisms underlying this association are not yet fully understood. Many studies have indicated that molecular mimicry could be the trigger for the development of certain diseases. However, as previously described, HTLV-1 can result in several immune response anomalies since it infects CD4^+^ T lymphocytes and alters their behavior (Figure 1) [65,66].

## 5. Rheumatoid Arthritis

RA is a chronic and incapacitating disease that affects 1% of the world’s population. Although the etiopathology of the disease is not fully understood, it is characterized by chronic polyarthritis that may lead to the destruction of articulation if not properly treated [67,68,69,70,71]. During the early stages of RA, the cytokines expressed in the synovium are mainly IL-2, 4, 13, 15, and 17. Once the disease is established, expression of IFN-γ, TNF-α, and IL-10 is observed, with low-level expression of IL-2, -4, -5, and -13. Furthermore, there is a correlation between serum cytokines and disease progression [72,73]. The development and progression of RA depends on the migration of the T lymphocytes into the synovium. Previous studies have observed proliferation of the synovium and T cell infiltration in HTLV infected patients that develop RA. Several studies reported the presence of HTLV proviral DNA in synovial liquid and tissue cells and that T cells in both the synovium and synovial cells were infected with HTLV-1. Nishioka *et al.* [74] also reported the expression of Tax mRNA in synovial cells from HTLV-I-associated arthropathy patients. Tax may induce cell proliferation, as well as the production of inflammatory cytokines. In addition, similar to what occurs in HAM/TSP, there is a migration of lymphocytes into the CNS. This migration may be associated with the viral load of the patient, as demonstrated by Yakova *et al.* [75]. These authors observed that patients infected with HTLV-1 that had RA or connective tissue disease had a higher viral load compared to asymptomatic patients; however, it was similar to the viral load of patients that developed HAM/TSP. Moreover, the viral load in the synovium was higher in RA patients [74,75,76,77,78,79].

## 6. Sjögren’s Syndrome

SS is defined as a systemic autoimmune disorder, manifesting primarily as xerostomia (dry mouth) and xerophthalmia (dry keratoconjunctivitis) due to the lymphocytic infiltration of the salivary and lachrymal glands, which in turn leads to the destruction of the ducts. Furthermore, antinuclear antibodies (ANA) and other self-antibodies, such as anti-SS-A (Ro) and SS-B (La), are also found in these patients. Disease development is also associated with genetic and hormonal factors [80,81]. Several viral infections, such as HTLV-1, may also be associated with the occurrence of this disease. A number of studies have reported a high prevalence of HTLV-1 in SS patients [82,83]. Nakamura *et al.* [84] reported a high prevalence of anti-HTLV-1 IgA in the salivary glands of SS patients. Another interesting related factor is the level of mononuclear infiltrate in SS patients infected with HTLV-1; SS patients with HTLV-1 show higher infiltrate levels compared with SS patients not infected with HTLV-1 [82,83,84].

## 7. Systemic Lupus Erythematosus

SLE is a systemic autoimmune disease of unknown etiology. This disease progresses with polymorphic clinical manifestations and periods of exacerbation and remission. Disease development is associated with a genetic predisposition and environmental factors, such as exposure to sunlight and viral infection [85,86,87,88].

The association between HTLV-1 and SLE is still controversial [89,90,91]. One possible mechanism proposed for this association is a process of molecular mimicry through the endogenous sequence related to HTLV-1 (HRES-1) in the development of SLE. This could trigger the production of self-antibodies, leading to the formation of immunocomplexes that are deposited in the tissues; this in turn could cause complement fixation and inflammation, which are pathogenic characteristics of SLE [92]. Other studies have demonstrated the expression of HTLV-1 antigens in the mononuclear cells present in the peripheral blood of individuals with SLE and infected with HTLV-1, following three or more days of *in vitro* culturing. This indicates the occurrence of viral replication in SLE patients, which could explain the high seropositivity for HTLV-1 and HTLV-2 observed in these patients [93].

## 8. Conclusions

Several studies have described HTLV-1 infection in the context of autoimmune diseases, although this subject is still under debate. Molecular mimicry has been hypothesized as a possible mechanism; however, HTLV-1-promoted altered cytokine release is critically involved in breaking tolerance. HTLV-1-induced changes in the activity of regulatory CD4 T-cell molecules affect the homeostasis of cytokines, including IFN- γ, TNF-α, TGF-β and IL-10, and disrupt the balance in inflammatory and anti-inflammatory responses, leading to the loss of tolerance and the development of autoimmunity.
